# Characteristics of Adopters of an Online Social Networking Physical Activity Mobile Phone App: Cluster Analysis

**DOI:** 10.2196/12484

**Published:** 2019-06-03

**Authors:** Ilea Sanders, Camille E Short, Svetlana Bogomolova, Tyman Stanford, Ronald Plotnikoff, Corneel Vandelanotte, Tim Olds, Sarah Edney, Jillian Ryan, Rachel G Curtis, Carol Maher

**Affiliations:** 1 Alliance for Research in Exercise, Nutrition and Activity School of Health Sciences University of South Australia Adelaide Australia; 2 Freemasons Foundation Centre for Men’s Health Faculty of Health Sciences The University of Adelaide Adelaide Australia; 3 Ehrenberg-Bass Institute School of Marketing University of South Australia Adelaide Australia; 4 School of Mathematical Sciences The University of Adelaide Adelaide Australia; 5 Priority Research Centre for Physical Activity and Nutrition University of Newcastle Newcastle Australia; 6 Physical Activity Research Group Central Queensland University Adelaide Australia

**Keywords:** k-medoid cluster analysis, social marketing, mobile phone app, physical activity, online social networking

## Abstract

**Background:**

To date, many online health behavior programs developed by researchers have not been translated at scale. To inform translational efforts, health researchers must work with marketing experts to design cost-effective marketing campaigns. It is important to understand the characteristics of end users of a given health promotion program and identify key market segments.

**Objective:**

This study aimed to describe the characteristics of the adopters of Active Team, a gamified online social networking physical activity app, and identify potential market segments to inform future research translation efforts.

**Methods:**

Participants (N=545) were Australian adults aged 18 to 65 years who responded to general advertisements to join a randomized controlled trial (RCT) evaluating the Active Team app. At baseline they provided demographic (age, sex, education, marital status, body mass index, location of residence, and country of birth), behavioral (sleep, assessed by the Pittsburgh Quality Sleep Index) and physical activity (assessed by the Active Australia Survey), psychographic information (health and well-being, assessed by the PERMA [Positive Emotion, Engagement, Relationships, Meaning, Achievement] Profile; depression, anxiety and stress, assessed by the Depression, Anxiety, and Stress Scale [DASS-21]; and quality of life, assessed by the 12-Item Short Form Health Survey [SF-12]). Descriptive analyses and a k-medoids cluster analysis were performed using the software R 3.3.0 (The R Foundation) to identify key characteristics of the sample.

**Results:**

Cluster analyses revealed four clusters: (1) younger inactive women with poor well-being (218/545), characterized by a higher score on the DASS-21, low mental component summary score on the SF-12, and relatively young age; (2) older, active women (153/545), characterized by a lower score on DASS-21, a higher overall score on the SF-12, and relatively older age; (3) young, active but stressed men (58/545) with a higher score on DASS-21 and higher activity levels; and (4) older, low active and obese men (30/545), characterized by a high body mass index and lower activity levels.

**Conclusions:**

Understanding the characteristics of population segments attracted to a health promotion program will guide the development of cost-effective research translation campaigns.

**Trial Registration:**

Australian New Zealand Clinical Trial Registry ACTRN12617000113358; https://www.anzctr.org .au/Trial/Registration/TrialReview.aspx?id=371463

**International Registered Report Identifier (IRRID):**

RR2-10.1186/s12889-017-4882-7

## Introduction

The risk of premature death caused by chronic diseases such as cardiovascular heart disease or diabetes has increased globally. Physical inactivity is a key risk factor for chronic disease, yet most adults in developed countries are insufficiently active to obtain health benefits [1,2]. Low cost, mass reach physical activity interventions are necessary to address the physical inactivity epidemic.

Technology-based interventions have become increasingly popular as the internet use has grown. It is estimated that internet use increased by 1052% between 2000 and 2018 with 54.4% of the world’s population now having internet access [3]. In addition to the increase in internet usage, online social media has become increasingly popular. Currently, Facebook has 2.2 billion active monthly users [4]. This has created opportunities for health behavior change interventions to be delivered via the internet and online social networks with more personalized and engaging features, potentially reaching a much larger population than previously possible.

Systematic reviews and meta-analyses suggest that online-delivered interventions can significantly increase physical activity [5-7]; however, relatively few such interventions are translated after the efficacy trial, and what studies find in randomized controlled trial (RCT) conditions may be different than the effectiveness of the same intervention in real-life conditions [8]. For example, the systematic review of Wu et al [9] of diabetes self-management apps found that only 1 out of 12 apps included in their meta-analysis was publicly available in the Apple or Android app stores. Another recent review of online self-help interventions found that just 30% were publicly available [10]. A number of barriers exist that hinder the translation of physical activity interventions developed and evaluated by researchers to the real world. This includes lack of resources to maintain or support software beyond the RCT, lack of resources to modify software designed for an RCT evaluation for wide-scale release, and lack of expertise and financial resources to promote the existence of the physical activity program so that it may be adopted by a large number of new users. A promising approach in terms of reach and cost effectiveness is to collaborate with marketers to translate health interventions at scale [11].

Social marketing uses commercial marketing approaches to influence positive behavior changes for individuals and communities [12,13]. Market segmentation analysis, also known as cluster analysis, is a social marketing technique used to identify homogeneous market segments of people who have similar needs or characteristics. Segments could be based on demographic, geographic, psychographic, or epidemiological factors. Identified segments can then be targeted with products or services designed or marketed specifically to address the segments’ needs, with the idea that such targeted products and services will be more appealing to specific population subgroups and therefore have higher adoption rates [14]. To date, few studies have used segmentation analysis in the context of adult physical activity. Friederichs et al [15] undertook a segmentation analysis based on physical activity motivational regulation of Dutch participants who had signed up to an eHealth (electronic health) physical activity intervention study. The researchers identified three groups (low motivation, controlled motivation, and autonomous motivation), and suggested that in the future, different intervention approaches might benefit individuals in different motivational clusters. Griffin et al [16] undertook a cluster analysis based on demographic and health behaviors (which included physical activity and other health behaviors such as smoking, alcohol consumption, and diet) of participants in a large Australian cohort study of older adults (the 45 and Up study). They identified six lifestyle behavior clusters, including one in which multiple risk behaviors clustered together, which they suggested highlighted the need for future targeted interventions to meet the needs of this group. Finally, Rundle-Thiele et al [13] undertook a market research study to identify social market segments based on physical activity behaviors, attitudes, and intentions in a sample of Australian adults. They identified four segments (young disinterested, successful enthusiasts, vulnerables, and happy retirees) and suggested that the study provided insights into key segments that could inform the development of future interventions. Thus, all three studies presented their findings as being potential targets for future intervention. However, to our knowledge, none has subsequently applied this information to directly inform the design or marketing of a real-world health promotion tool.

This study aimed to use a similar approach as previous segmentation studies to directly inform the development of a marketing strategy to promote an evidence-based online social networking physical activity program [17]. Active Team is a gamified, online social networking physical activity intervention that aims to increase physical activity by encouraging users to undertake a 100-day physical activity challenge with their friends [17]. The program incorporates social influence and gamification techniques and is used in conjunction with a step-counter. In collaboration with marketing experts, we are currently planning a translation study that will attempt to disseminate the program widely using an online social marketing campaign. This study aimed to inform the translational study by (1) examining the characteristics of adopters of the Active Team program and (2) identifying clusters among the adopters based on sociodemographic, psychographic, and behavioral characteristics. Findings will be useful to inform software improvements and develop segmented marketing strategies when Active Team is disseminated to the public.

## Methods

### Statement of Ethics

Ethics approval for this study was provided by the University of South Australia’s Human Research Ethics Committee (protocol number 0000033967), and all participants provided informed consent. This study was undertaken in the context of an RCT that ran from late 2016 to late 2018 [17].

### Participants and Data Collection

Participants were Australian adults aged 18 to 65 years recruited between October 2016 and February 2017 to participate in an RCT evaluating a social and gamified physical activity intervention entitled Active Team. Promotional efforts for the Active Team app and the research trial evaluating it included mainstream media news stories, flyers, and paid, nontargeted Facebook advertisements, with the vast majority of participants signing up via Facebook rather than the other promotional methods. The Facebook advertisements featured still images rather than videos, accompanied by text captions, and were designed to be gender neutral (ie, images of both men and women). The advertisements were set so that they would be shown to men and women aged 18 to 65 years located anywhere in Australia, with no other targeting. The ads were developed in consultation with an online marketing academic (Professor Karen Nelson-Field). From March 1, 2017, targeted recruitment efforts were commenced in an effort to increase enrollment of men into the study. Therefore, this study only includes the participants recruited through nontargeted advertisements, up to the end of February 2017.

Interested adults were directed to download the app from the Apple and Google Play App Stores before enrolling. The app contained a feature that allowed them to send online invitations to their friends who could then also register their interest. Potential participants were screened for initial eligibility (aged between 18 to 65 years, insufficiently active, use of Facebook at least weekly, fluent in English, and living within Australia) and asked to provide informed consent and complete a baseline questionnaire. Participants were required to use Facebook weekly since the Active Team software itself integrated with Facebook by connecting users to their Facebook friends within the Active Team app. More detail regarding the RCT is available in the protocol paper [17].

Following this initial phase, to be formally enrolled in the study, participants needed to successfully wear an accelerometer for one week and have at least two friends successfully complete the baseline assessments (including accelerometry). However, for inclusion in our analysis, participants only needed to pass initial screening, provide informed consent, and complete the baseline survey. By focusing on adopters, we aimed to gain insights into those groups the app is likely to appeal to the most.

The baseline questionnaire was administered via Qualtrics online survey software and captured demographic information including residential address, date of birth, sex, marital status, height and weight (to calculate body mass index [BMI]), country of birth, and highest education level.

Self-reported weekly minutes of moderate to vigorous physical activity was collected using the Active Australia Survey. This 8-item questionnaire captures walking and other physical activities undertaken in both leisure and household duty contexts including the intensity of the activity in the preceding week [18]. To calculate sufficient activity, the time spent walking or in moderate activity and twice the time spent in vigorous activity were summed. Sufficient activity was interpreted as a total of at least 150 minutes of activity per week. The survey has reasonable test-retest reliability (intraclass correlation coefficient .52) [19] relative to accelerometry (*r*=.49-.64) [20].

Self-reported quality of life was measured using the validated 12-Item Short Form Health Survey (SF-12). This scale assesses both physical and mental quality of life domains [21]. Summary scales were scored using US population norms creating two measures for physical and mental component summary scores. The survey’s test-retest reliability (two weeks apart) was .89 for the physical component summary and .76 for the mental component summary [22]. Validity of the SF-12 physical and mental component summary scores was correlated with the physical component summary-36 (*r*=.951) and mental component summary-36 (*r*=.969) equating to an *R*^2^ of .904 for the physical component summary and an .939 for the mental physical component summary [22].

Well-being was assessed using the Positive Emotion, Engagement, Relationships, Meaning, Achievement (PERMA) Profile measure. This 23-item scale measures well-being across well-being pillars (positive emotion, engagement, relationships, meaning, and accomplishment) using 11-point Likert-type items (0=not at all to 10=completely) [23]. The PERMA Profile measure for overall well-being was scored by calculating the mean for all 23 items [23]. Test-retest reliability (two weeks apart) was .80 for accomplishment, .86 for meaning, .83 for relationships, .78 for engagement, and .84 for positive emotions [23]. Validity correlation coefficients were .79 when compared with the Satisfaction with Life Scale, and .87 when compared with Flourishing Scale [23].

Depression, anxiety and stress were measured using the Depression and Anxiety Stress Scale (DASS-21). This scale comprises 21 questions using a 4-point Likert-type scale (0=not at all, 3=almost always) [24]. The DASS-21 was scored by summing the scores for depression, anxiety, and stress then multiplying the sums by two as the original version of the DASS has 42 items [24]. Test-retest reliability (three weeks apart) was .77 (95% CI .56-.88) for depression, .89 (95% CI .81-.94) for anxiety, and .85 (95% CI .51-.94) for stress [25]. Validity correlation coefficients were .79 for the depression scale when compared with Beck Depression Inventory, .85 for the anxiety scale when compared with Beck Anxiety Inventory, and .68 for the stress scale when compared with State-Trait Anxiety Inventory–Trait Version [26].

Sleep quality and quantity were collected using the Pittsburgh Sleep Quality Index (PSQI). Self-report sleep duration was recorded in minutes, and sleep quality was measured on a 4-point Likert-type scale (1=very bad, 2=bad, 3=good, and 4=very good) [27]. The results were then presented as categorical data in the analysis and presented as total count and percentage of the sample. Test-retest reliability and validity were obtained: a score greater than 5 resulted in sensitivity of 89.6% and specificity of 86.5% (kappa=.75, *P*<.001) [27].

Participant goal-setting behavior, outcome expectations, self-efficacy, and intentions were assessed using the 21-item Social Cognitive Theory (SCT) scale [28-31]. A composite score for each variable was calculated by taking the mean of items for that variable. Test-retest reliability and validity were established for the decisional balance scales [28].

### Selection of Cluster Inputs

The questionnaire items described above produced a total of 29 possible cluster inputs. However, the number of variables that can be used in a cluster analysis depends on the sample size. Formann [32] recommends that the minimal sample size should be 2^k^, where k represents the number of variables. The number of cases with complete data was 459, therefore eight variables could be used in the cluster analysis. To determine which eight variables should be included, we first produced a correlation matrix to determine whether any of the potential cluster inputs were collinear. No collinearity was detected. Therefore, the cluster inputs were decided by discussion among the authors who represented a wide range of academic disciplines including health sciences, behavioral science, and marketing based on the following parameters: first, we prioritized variables that could be used to deliver targeted online advertising (eg, age, sex). Second, we prioritized variables that represented ancillary benefits of physical activity that could be used in marketing messaging such as stress or BMI (eg, for people with high levels of stress, the physical activity program could be marketed by highlighting the positive role of physical activity in managing stress) [33]. Third, we aimed to maximize variety in the types of included outcomes (eg, a range of sociodemographic, physical, behavioral, and psychological variables) to gain further insight and potential marketing strategies from the sample. As a result, the following eight cluster input variables were chosen: sex, age, physical activity level, education, BMI, overall stress, overall well-being, and physical activity self-efficacy.

### Analysis

Descriptive statistics were inspected for all study variables. Continuous variables were examined for normality. The sample was described in terms of means and standard deviations. Categorical variables such as sex were described in terms of total count and percentage of the sample.

K-medoids cluster analyses were performed to identify segments within the sample. This cluster analysis approach was selected on the basis that it permits analysis of categorical data [34,35], can handle nonnormally distributed variables, and has been previously used in health-related market segmentation research [15]. In addition, it is more robust to noise and outliers than the k-means approach [34]. Analyses, conducted with R 3.3.0 (The R Foundation), used the partitioning around medoids (PAM) algorithm with the Gower metric. The analysis finds data points or medoids within the data whose average dissimilarity to all the objects in the cluster is minimized. It begins by finding an initial set of medoids, then iteratively replaces one medoid by one nonmedoid until it determines best fit [36]. Analyses were set to produce solutions for between two to eight clusters on the basis that this number of clusters allowed segmentation that would result in meaningful and interpretable clusters [34]. The optimal number of clusters was subsequently determined by identifying the cluster solution that resulted in the maximum average silhouette width. The influence of multivariate outliers on the cluster solution was examined by plotting the squared Mahalanobis distances of the principal components against the empirical chi-squared distribution and identifying data points beyond the 97.5 percentile. Sixteen data points were considered outliers for females and four for males. Outliers were removed, and the data were reclustered. Cohen kappa between cluster solutions with and without outliers showed high agreement (.86 females and .64 for males); therefore, the outliers were retained.

Cluster stability of the final models was examined by randomly generating 99 subsamples of 80% of the full sample and computing the average Rand index for the cluster solutions, using the R package clv [37]. The Rand index indicates the proportion of pairs of cluster allocations that agree between the full sample and the subsample, resulting in a value between 0 (complete disagreement) and 1 (complete agreement) [38].

## Results

### Participant Characteristics

The demographic details of the 484 participants are summarized in Table 1. In brief, the majority were female (392/484, 81.0%), living with a partner (348/484, 71.9%), had a university education (275/484, 56.8%), and lived in a major city (455/484, 94.0%). The average age was 41 years, and the majority were overweight or obese (≥25, 78%). Compared with available Australian population data, participants who signed up to use the online social networking physical activity program were more likely to be female (81% vs 51%), cohabit with a partner (72% vs 58%), have a university education (57% vs 22%), be born in Australia (72% vs 67%) [39], live in a major city (94% vs 69%) [40], and be overweight or obese (78% vs 63%) [41]. At the group level, physical and mental component summary scores were within the normal range for the SF-12 [21]. However, depression, anxiety and stress were relatively high compared with previously published Australian population data (depression mean 4.8 [SD 5.1] vs 2.6 [SD 3.9], anxiety mean 3.6 [SD 3.8] vs 1.7 [SD 2.8], and stress mean 7.5 [SD 4.9] vs 4.0 [SD 4.2]) [42].

### Cluster Analysis

Two cluster analysis models were performed on a total of 459 participants who had complete data for the variables selected for inclusion in the analysis. An initial cluster analysis resulted in trivial clusters determined entirely by sex and educational level. Therefore, we removed education, stratified the population by sex, and performed separate cluster analyses for men and women using six cluster inputs due to the reduced sample by separating for sex. The silhouette widths were maximized when two clusters were derived for each sex (Table 2).

**Table 1:** Descriptive characteristics of the study sample (n=484).

Characteristics		Value
Age in years, mean (SD)		40.8 (12.1)
Sex, female, n (%)		392 (81.0)
**Marital status, n (%)**		
	Partnered	348 (71.9)
	No partner	121 (25.0)
	Prefer not to disclose	15 (3.1)
Body mass index, mean (SD)		30.1 (6.7)
**Education, n (%)**		
	High school or lower	73 (15.1)
	Some post-high school (eg, trade or diploma)	136 (28.1)
	University	275 (56.8)
Australian-born, n (%)		348 (71.7)
**ASGS^a^ remoteness, n (%)**		
	Major city	455 (94.0)
	Inner regional	19 (3.9)
	Outer regional	5 (1.0)
	Remote	5 (1.0)
	Very remote	0 (0)
**Social cognitive theory, mean (SD)**		
	Goal setting (scale range 1-5)	3.0 (0.1)
	Outcome expectations (scale range 1-5)	3.3 (0.4)
	Intention (scale range 1-7)	4.1 (0.7)
	Self-efficacy (scale range 1-5)	3.0 (0.7)
Positive Emotion, Engagement, Relationships, Meaning, Achievement, mean (SD) (scale range 1-10)		6.5 (1.5)
**12-Item Short Form Health Survey, mean (SD) (scale range 0-100)**		
	Physical component summary	40.3 (6.1)
	Mental component summary	48.0 (9.4)
**PSQI^b^ sleep quality, n (%)**		
	Very good	24 (5.0)
	Good	223 (46.1)
	Bad	198 (40.9)
	Very bad	39 (8.1)
**Depression, Anxiety, and Stress Scale, mean (SD)**		
	Depression	4.8 (5.1)
	Anxiety	3.6 (3.8)
	Stress	7.5 (4.9)

^a^ASGS: Australian Statistical Geography Standard [43].

^b^PSQI: Pittsburgh Sleep Quality Index.

**Table 2:** Female and male clusters sex, age, physical activity level, body mass index, stress, well-being, and self-efficacy per cluster.

Characteristic	Cluster 1 (n=218)	Cluster 2 (n=153)	Cluster 3 (n=58)	Cluster 4 (n=30)
Sex, male, n (%)	0 (0)	0 (0)	58 (100)	30 (100)
Age, mean (SD)	33.4 (7.8)	52.8 (5.9)	30.7 (7.7)	52.5 (6.5)
Weekly minutes PA^a^, mean (SD)	226 (282)	204 (233)	297 (296)	159 (183)
Body mass index, mean (SD)	29.6 (7.5)	30.9 (6.4)	28.4 (5.5)	33.1 (4.3)
Stress, mean (SD)	8.4 (4.7)	6.0 (4.1)	8.9 (6.4)	6.2 (4.5)
Well-being, mean (SD)	6.4 (1.5)	6.6 (1.7)	6.3 (1.5)	6.7 (1.5)
Self-efficacy, mean (SD)	3.0 (0.7)	2.9 (0.7)	3.0 (0.8)	3.3 (0.9)

^a^PA: physical activity.

Among women, cluster 1 consisted of relatively younger inactive women with high stress, while cluster 2 was characterized by older women with lower stress and higher physical activity. Among men, cluster 3 was characterized by younger, stressed but physically active men, while cluster 4 consisted of older, inactive, and predominantly obese men. The median Rand index was 0.87 (IRQ 0.62-1.00) for the female cluster solution and 0.75 (IRQ 0.58-0.87) for the male cluster solution.

**Figure 1:**
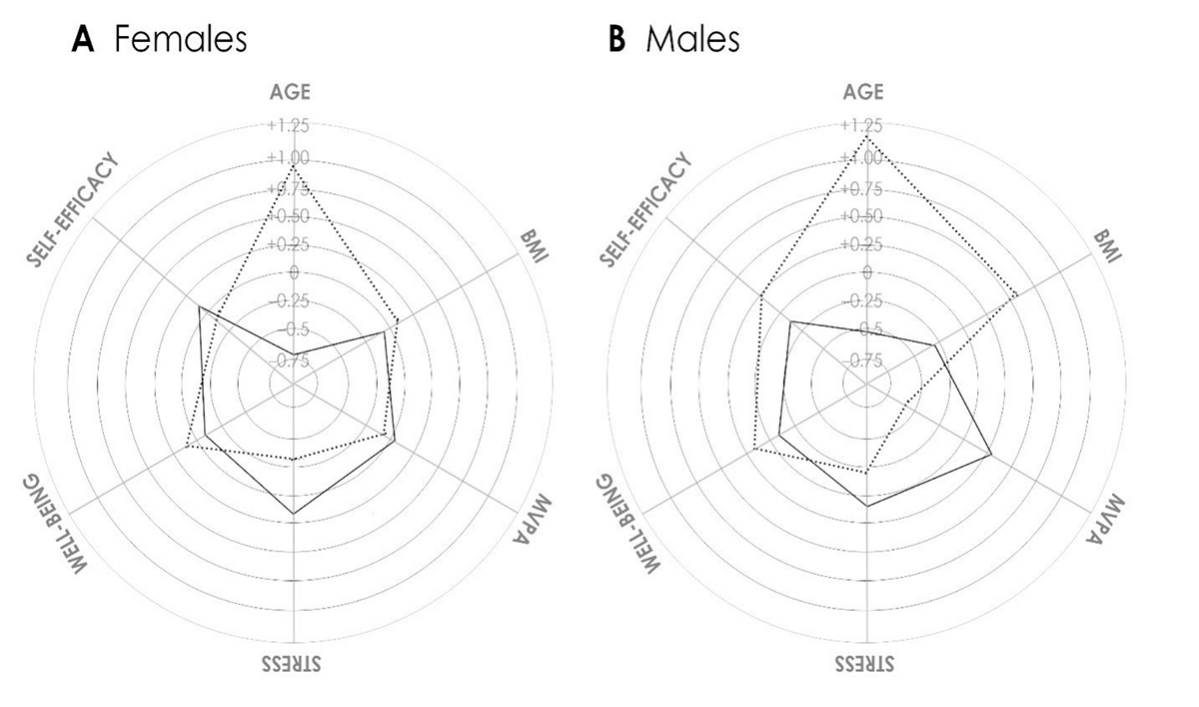
Radar graphs of the female and male cluster solution. BMI: body mass index, MVPA: moderate and vigorous physical activity.

Clinical differences (eg, psychosociodemographic characteristics) of the clusters other than cluster inputs were then examined (Table 3). Women in cluster 1 were more likely to be single, have lower mental quality of life, and report higher depression and anxiety than women in cluster 2. Among the men, those in cluster 3 were more likely to be single compared with men in Cluster 4. The cluster groups are presented in Figure 1.

**Table 3:** Descriptive characteristics of the clusters.

Characteristic		Cluster 1 (n=218)	Cluster 2 (n=153)	Cluster 3 (n=58)	Cluster 4 (n=30)
**Marital status, n (%)**					
	Partnered	146 (67.0)	121 (79.1)	32 (55.2)	28 (93.3)
	No partner	61 (28.0)	28 (18.3)	25 (43.1)	2 (6.7)
	Prefer not to disclose	11 (5.0)	4 (2.6)	1 (1.7)	0 (0)
**Education, n (%)**					
	High school or lower	28 (12.8)	20 (13.1)	13 (22.4)	8 (26.7)
	Some post-secondary	59 (27.1)	46 (30.1)	12 (20.7)	6 (20.0)
	University	131 (60.1)	87 (56.9)	33 (56.9)	16 (53.3)
Australian-born, n (%)		181 (83.0)	127 (83.0)	39 (67.2)	26 (86.7)
**Social Cognitive Theory, mean (SD)**					
	Goal setting	3.1 (1.0)	2.9 (1.0)	2.9 (1.0)	2.8 (0.9)
	Outcome expectations	3.3 (0.4)	3.3 (0.4)	3.4 (0.4)	3.3 (0.4)
	Intention	4.1 (0.7)	4.0(0.6)	3.9 (0.8)	4.2 (0.7)
**12-Item Short Form Health Survey, mean (SD)**					
	Physical component	40.2 (5.8)	40.3 (6.4)	40.5 (6.1)	38.9 (7.0)
	Mental component	46.6 (10.0)	50.3 (8.4)	46.8 (9.0)	48.7 (9.7)
**Pittsburgh Sleep Quality Index, n (%)**					
	Very good	13 (6.0)	8 (5.2)	4 (6.9)	0 (0)
	Good	105 (48.2)	70 (45.8)	25 (43.1)	14 (46.7)
	Bad	87 (39.9)	64 (41.8)	23 (39.7)	12 (40.0)
	Very bad	13 (6.0)	11 (7.2)	6 (10.3)	4 (13.3)
**Depression, Anxiety, and Stress Scale, mean (SD)**					
	Overall depression	5.2 (5.0)	3.9 (4.8)	6.0 (5.6)	4.2 (5.5)
	Overall anxiety	3.9 (3.8)	2.7 (3.1)	5.0 (5.2)	3.0 (3.5)

## Discussion

### Principal Findings

This study aimed to determine the characteristics of adopters of an online social networking physical activity intervention and use segmentation analysis to identify homogenous market segments of users. Results revealed that the online social networking physical activity intervention attracted people who were well educated, urban, and female and had a higher BMI and higher depression, anxiety, and stress levels compared with population norms. The segmentation analysis identified four clusters within the adopters: younger inactive women with relatively poor mental well-being, older physically active women, younger active but stressed men, and older obese and inactive men.

The fact that the study attracted mostly urban residents, females, and people with a university education is consistent with previous health promotion research. For example, Duggan and Brenner [44] similarly reported that women and those with a higher level of education are more likely to engage with social media and online health interventions. In addition, a systematic review examining the effectiveness of online social networks to improve health behaviors conducted by Maher et al [6] reported high female participation rates (83.3%). It is also common for RCTs to have more difficulty engaging with rural participants [45]. People living in rural and remote areas are recognized to be underserved in terms of primary prevention and health care access. Online interventions appear to be a good avenue for addressing such access inequalities; however, our results suggest that access barriers remain. This could be because despite the avenue of delivery women are generally more engaged with their health and well-being [44].

In general, the key traits that determined cluster membership were age, sex, physical activity, BMI, and stress. This is reasonably consistent with previous studies of this nature with the variables age, physical activity, and BMI consistently appearing in cluster memberships with physical activity and BMI being age-related [13,15]. This study differed from previous research in that we included more psychographic measures. Cluster membership was segmented based on stress in both female and male clusters. In addition, among women, there were significant differences between the clusters for the SF-12 mental component and the depression and anxiety scales for the DASS-21. It is possible that power was an issue when attempting to identify statistical differences between the men’s clusters relative to the women’s.

### Strengths and Limitations

Before considering the implications of these findings, study strengths and weaknesses should be acknowledged. To the best of the authors’ knowledge, no technology-based physical activity programs to date have used segmentation analysis to inform translation efforts. Our approach was highly interdisciplinary, with the team including health, marketing, and statistical experts. We were able to use a wide range of outcome measures (sociodemographic, behavioral, psychographic, and physical), providing insights across many potential marketing targets. Furthermore, a statistical strength of this study was that we used the PAM algorithm with Gower metric, which improves the strength and validity of the clusters compared with conventional cluster analysis methods.

This study also has some limitations. First, due to the sample size, the number of cluster inputs had to be restricted to eight and then six. It is possible that choosing different cluster inputs may have resulted in different clusters. In addition, the sample may limit generalization of our findings to other contexts. Specifically, despite using a nontargeted advertising approach, it is possible that the participant demographics were influenced by the recruitment strategies used and that a different advertising approach may have resulted in a different sample. Additionally, participants registered interest in an RCT of the online social networking intervention, and it is possible that this group is different from people who may download and use the app in a real-life situation. However, it is difficult to postulate in what ways they would differ—for example, it is possible that the RCT may attract relatively highly motivated users (since they are willing to take on the assessment burden associated with research participation). Conversely, it is possible that the RCT actually attracts less motivated people—for example, if they are motivated by a financial incentive offered by a study or are aware they lack intrinsic motivation and so are seeking the imposition of external discipline. Despite these limitations, this approach enabled us to examine a wider variety of participant characteristics than would be possible by examining users of a commercial app, providing valuable information for disseminating similar apps via online advertising in the future.

### Implications

Technology-based health promotion using mobile phones is a growing field in research with great potential to impact the health and well-being of many. To date, however, relatively few evidence-based interventions developed and tested in research settings are ever attempted to be translated to the real world [7]. Successful translation will require complementary approaches that go beyond RCT designs to understand the true impact of public health interventions on the general population in everyday conditions.

The market segments identified in this study as being more likely to be attracted to this type of physical activity program used in a research setting were younger low-active women with poor mental well-being, older active women, young active but stressed men, and older low-active and obese men. All segments comprised people of relatively high educational status, with the majority being university educated. Such information may be used to inform future media and communication channels, given that media usage patterns differ by demographic segment. For example, an Instagram campaign may be useful to reach the young female cluster identified in the segment analysis, given that approximately 60% of Instagram users are aged between 18 and 35 years and the platform is equally popular for men and women [46].

The psychographic characteristics of the identified clusters provide guidance for the potential benefits people in these segments may be seeking by joining the program. These benefits (or solutions to their existing problems) can be used to inform marketing messages and translate those messages during promotional campaigns for physical activity programs. As an example, given that a major cluster comprised younger women with relative low well-being, marketing campaigns may focus on the positive effects physical activity has on mental health [33], or alternatively, suggest that additional program features targeted at improving well-being may be warranted.

The analysis also highlighted that this app largely did not reach certain demographics—for example, men and people of low educational status were underrepresented. This raises the possibility of two very different directions for future in-market program promotion efforts. The first option is to play to the program’s current strengths and tailor marketing efforts for the ecological trial to the adopter market segments identified in this study. This approach could be expected to result in higher return on investment—that is, higher participation rate from the specific targeted groups (higher participation from the stressed younger women and almost no participation from other nontargeted segments). An alternative approach would be to go after a mass market including people who were not captured in this adopter study. While some of these segments might be much harder to reach (eg, males), keeping the approach and message more general might result in lower overall numbers of enrollments into the health program. The utility and cost effectiveness of each of these approaches can be tested in a well-planned ecological trial.

### Conclusion

Technology-based health promotion programs offer great promise for delivering effective, appealing, and accessible interventions. However, for their full potential to be reached, evidence-based interventions must be scaled up, in particular to reach audience segments that health behavior research does not typically reach (eg, men, those of low socioeconomic status). This requires multidisciplinary efforts, teaming health researchers with marketers. This study serves as a novel example of how research translation might proceed following an RCT and demonstrates how a social marketing framework and market segmentation analysis approach may provide insights to guide future software improvements and targeted marketing efforts. Such judicious, evidence-based approaches are likely to be helpful, given that health researchers seek to deliver the greatest impact with limited program development and marketing budgets. Additional research aimed at identifying clusters of adopters of health apps in larger samples from more ecologically valid contexts would provide an important contribution to this emerging literature and further assist health researchers planning dissemination of health promotion programs.

## Abbreviations

BMI: body mass index
DASS-21: Depression, Anxiety, and Stress Scale
eHealth: electronic health
PAM: partitioning around medoids
PERMA: Positive Emotion, Engagement, Relationships, Meaning, Achievement
PSQI: Pittsburgh Sleep Quality Index
RCT: randomized controlled trial
SCT: Social Cognitive Theory
SF-12: 12-Item Short Form Health Survey
